# Activation of TRPV4 by mechanical, osmotic or pharmaceutical stimulation is anti-inflammatory blocking IL-1β mediated articular cartilage matrix destruction

**DOI:** 10.1016/j.joca.2020.08.002

**Published:** 2021-01

**Authors:** S. Fu, H. Meng, S. Inamdar, B. Das, H. Gupta, W. Wang, C.L. Thompson, M.M. Knight

**Affiliations:** Centre for Predictive *In Vitro* Models, School of Engineering and Materials Science, Queen Mary University of London, UK

**Keywords:** Cartilage, IL-1β, TRPV4, Mechanotransduction, Hypo-osmolarity, Cilia

## Abstract

**Objective:**

Cartilage health is maintained in response to a range of mechanical stimuli including compressive, shear and tensile strains and associated alterations in osmolality. The osmotic-sensitive ion channel Transient Receptor Potential Vanilloid 4 (TRPV4) is required for mechanotransduction. Mechanical stimuli inhibit interleukin-1β (IL-1β) mediated inflammatory signalling, however the mechanism is unclear. This study aims to clarify the role of TRPV4 in this response.

**Design:**

TRPV4 activity was modulated glycogen synthase kinase (GSK205 antagonist or GSK1016790 A (GSK101) agonist) in articular chondrocytes and cartilage explants in the presence or absence of IL-1β, mechanical (10% cyclic tensile strain (CTS), 0.33 Hz, 24hrs) or osmotic loading (200mOsm, 24hrs). Nitric oxide (NO), prostaglandin E_2_ (PGE_2_) and sulphated glycosaminoglycan (sGAG) release and cartilage biomechanics were analysed. Alterations in post-translational tubulin modifications and primary cilia length regulation were examined.

**Results:**

In isolated chondrocytes, mechanical loading inhibited IL-1β mediated NO and PGE_2_ release. This response was inhibited by GSK205. Similarly, osmotic loading was anti-inflammatory in cells and explants, this response was abrogated by TRPV4 inhibition. In explants, GSK101 inhibited IL-1β mediated NO release and prevented cartilage degradation and loss of mechanical properties. Upon activation, TRPV4 cilia localisation was increased resulting in histone deacetylase 6 (HDAC6)-dependent modulation of soluble tubulin and altered cilia length regulation.

**Conclusion:**

Mechanical, osmotic or pharmaceutical activation of TRPV4 regulates HDAC6-dependent modulation of ciliary tubulin and is anti-inflammatory. This study reveals for the first time, the potential of TRPV4 manipulation as a novel therapeutic mechanism to supress pro-inflammatory signalling and cartilage degradation.

## Introduction

Osteoarthritis (OA) effects over 4.4 million people in the UK alone representing significant economic cost^1^. Cartilage health is maintained in response to mechanical stimuli, articular cartilage is routinely exposed to a wide array of dynamic mechanical loading consisting of compressive, shear and tensile strains as well as associated alterations in fluid shear and osmolality^2^. Mechanical loading in the form of compression or tensile strain is anti-inflammatory in chondrocytes and blocks the release of the pro-inflammatory mediator's nitric oxide (NO) and prostaglandin E_2_ (PGE_2_) in response to interleukin-1β (IL-1β)^3^, ^4^, ^5^. Inflammatory signalling contributes to cartilage degradation in OA thus understanding the link between mechanical loading and inflammation will have significant therapeutic impact.

The osmotic-sensitive Ca^2+^ ion channel Transient Receptor Potential (TRP) Vanilloid 4 (TRPV4) is highly expressed in articular chondrocytes and is activated by mechanical stimuli^6^^,^^7^. TRPV4 is required for mechanotransduction in chondrocytes and other cells types^7^, ^8^, ^9^. It mediates the regulation of pro-anabolic and anti-catabolic genes promoting matrix production and accumulation in agarose-embedded chondrocytes^7^^,^^9^. TRPV4 mutations result in human skeletal dysplasia suggesting a role in bone development (for review see^10^). Indeed, chondrocytes from TRPV4^−/−^ mice exhibit loss of osmosensitivity accompanied by joint degeneration indicating a central role for this channel protein in maintaining joint homeostasis^11^^,^^12^. Pharmaceutical activation of TRPV4 inhibits NO release in response to inflammatory cytokines suggesting a potential mechanistic role in the anti-inflammatory effects of mechanical loading^13^^,^^14^. However, in apparent contradiction of these findings TRPV4 inhibition exerts an anti-inflammatory effect in the cardiovascular system, lung and peripheral nervous system^15^, ^16^, ^17^. This study therefore aims to clarify the regulatory role of TRPV4 in cartilage inflammatory signalling.

TRPV4 localises to the plasma membrane and primary cilium, a small microtubule based signalling compartment present at the cell surface^18^^,^^19^. Primary cilia have been implicated in both chondrocyte mechanotransduction^20^, ^21^, ^22^ and inflammatory signalling^23^, ^24^, ^25^, ^26^. The cytoplasmic tubulin deacetylase histone deacetylase 6 (HDAC6) is enriched within the cilium and modulates cilia resorption through de-acetylation and polymerization of ciliary tubulin^27^, ^28^, ^29^. Post translational modification of ciliary tubulin influences intraflagellar transport (IFT), the microtubule based motility present within the cilium required for cilia-mediated signalling^30^^,^^31^. Previously we report that mechanical loading counteracts inflammatory signalling in response to the pro-inflammatory cytokine interleukin 1β (IL-1β) via HDAC6 activation in association with alterations in IFT/cilia^5^. A role for TRPV4 in this pathway has not previously been identified.

In the present study, we demonstrate for the first time that TRPV4 activation by cyclic tensile strain (CTS), hypo-osmotic challenge or the TRPV4 agonist GSK1016790 A inhibits pro-inflammatory IL-1β signalling and cartilage degradation associated with alterations in primary cilia elongation. TRPV4 may therefore provide a novel target for the treatment of joint disease and other inflammatory pathologies.

## Methods

### Antibodies and reagents

Chondrocytes were treated with interleukin-1β (IL-1β, 200-01 B; Peprotech, London, UK), TRPV4 antagonist GSK205 (616,522; Merck Millipore, London, UK) and agonist GSK1016790 A (GSK101, G0798; Sigma Aldrich, Poole, UK). Antibodies for immunocytochemistry: acetylated α-tubulin (1:2,000, T7451, Sigma Aldrich, Poole, UK) and TRPV4 (1:200, SAB2104243, Sigma Aldrich). Nuclei were counterstained with 4′,6-diamidino-2-phenylindole (4′,6-diamidino-2-phenylindole (DAPI), Sigma Aldrich). Antibodies for western blotting: acetylated α-tubulin (1:1,000, T7451, Sigma Aldrich) and α-tubulin (1:1,000, ab4074, Abcam, Cambridge, UK).

### Cartilage explant and chondrocyte culture

Bovine cartilage explants and chondrocytes were obtained from 16 month steers as previously described^28^. Full depth articular cartilage was removed from the proximal surface of the metacarpal phalangeal joint and chondrocytes isolated by enzymatic digestion. Explants were harvested using a 5 mm diameter biopsy punch (BP–50 F, Selles Medical Ltd, UK). Both were cultured at 37 °C, 5% CO_2_ in Dulbeccos Minimal Essential Medial (DMEM, D5921, Sigma–Aldrich, Poole, UK) supplemented with 10% (v/v) foetal calf serum (FCS, F7524, Gibco, Paisley, UK), 1.9 mM l-glutamine (G7513), 96 U/ml penicillin (P4333, All Sigma–Aldrich, Poole, UK). Explants were rested for 2 d prior to experimentation while isolated chondrocytes were cultured to confluence.

### Application of cyclic tensile strain

Isolated chondrocytes were cultured on collagen coated flexible elastomeric membranes and subjected to uniform, equibiaxial CTS applied using the Flexcell 5000 T system (Dunn Labortechnik GMbH). Cells were subjected to 0–10% strain for 24 h at 0.33 Hz.

### Application of osmotic loading

Isolated cells were cultured for 24 h without serum in osmotically adjusted media at 200, 315 or 400 mOsm, hereafter referred as hypo-, iso- or hyper-osmotic media respectively. Explants were cultured for up to 12 d under similar conditions with the addition of serum to maintain chondrocyte viability resulting in a slightly higher osmolarity of 318 mOsm for the iso-osmotic media. The osmolarity of all solutions was adjusted by adding D-mannitol (M4125, Sigma–Aldrich) or distilled water and measured using a freezing point depression osmometer.

### Biochemical analysis of NO, PGE_2_ and sGAG release

Nitric Oxide (NO) release was assessed using the Griess assay based on quantification of nitrite (NO_2_), the stable product of NO degradation. Nitrite content was quantified against a sodium nitrite standard curve using the Galaxy Fluorstar spectrophotometer (BMG Labtech, UK). An immunoassay kit (KGE004B, R&D Systems, UK) was used to quantify PGE_2_ concentrations in the media according to the manufacturer's instructions. Results were corrected for non-specific binding and calibrated using a PGE_2_ standard curve. The release of sulphated glycosaminoglycan (sGAG) into the culture media was quantified using the dimethylmethyleneblue (DMMB) assay against a chondroitin sulphate standard curve (6-sulphate:4-sulphate; 0.33:1; Sigma–Aldrich).

### Immunocytochemistry, live imaging and confocal microscopy

For immunocytochemistry, samples were fixed with 4% paraformaldehyde for 10 min, permeabilised for 5 min with 0.5% triton-X100/phosphate buffered saline (PBS) then blocked with 5% goat serum/PBS for 1 h. Primary antibody was incubated at 4°C overnight followed by appropriate Alexa Fluor conjugated secondary antibodies (Molecular Probes) for 1 h at room temperature. Cells were counterstained with 1 μg/ml 4',6-diamidino-2-phenylindole (DAPI) for 5 min. For live imaging, cell viability was assessed by live/dead staining. Explants were incubated for 30 min with 5 μM Calcein acetyl methyl (AM) and 5 μM Ethidium homodimer-1 (EthD-1) prepared in appropriate osmotic adjusted media, washed and immediately imaged. Samples were imaged using a Zeiss 710 ELYRA PS.1 microscope. For cilia analysis, samples were imaged using an x63/1.4 numerical aperture (NA) objective to generate confocal z-sections made throughout the cell depth (approximately 20 sections) using 0.25 μm step size with an image format of 1,024 x 1,024 yielding a pixel size of 0.13 × 0.13 μm (image size approximately 135 × 135 μm). Cilia length and prevalence was quantified from resulting maximum projection images using Image J software (National Institutes of Health, Maryland, USA).

### Western blotting

Cells were lysed in radioimmunoprecipitation (RIPA) buffer (R0278, Sigma Aldrich) and total protein quantified by Bicinchoninic acid (BCA) assay. For the fractionation of soluble and polymerized tubulin, extraction buffer A (137 mM NaCl, 20 mM Tris–HCl, 1% Triton X-100, and 10% glycerol) was added to cells at 4 °C for 3 min, plates were gently swirled and the buffer removed and saved as the soluble tubulin fraction. Immediately after, extraction buffer B (buffer A + 1% sodium dodecyl sulphate (SDS)) was added for 1 min, the remaining sample was scraped, incubated on ice for 30 min and saved as the polymerized tubulin fraction.

SDS–PAGE was performed under reducing conditions and proteins transferred to nitrocellulose membranes. Membranes were blocked in odyssey blocking buffer (Li-Cor Cambridge, UK) prior to overnight incubation with primary antibodies and infrared secondary antibodies (Li-Cor). Proteins were visualized using the Li-Cor Odyssey and quantified using Image Studio Lite software (Li-Cor).

### HDAC6 activity measurement

A commercial fluorometric assay kit (K466-100, Biovision) was used to measure HDAC6 activity according to the manufacturer's instructions. This assay determines enzyme activity by exploiting the selectivity of tubacin for HDAC6 in combination with a fluorescent synthetic acetylated-peptide substrate. Cultures were lysed, a 10 μl aliquot was mixed with either acetylated substrate (sample) or with 2 μM tubacin and acetylated substrate (inhibitor control) then incubated for 30 min at 37°C. The deacetylase-dependent release of a 7-amino-4-trifluoromethylcoumarin fluorophore (excitation/emission at 350/490 nm) was then measured on a Galaxy Fluorstar spectrophotometer (BMG Labtech, UK) and HDAC6 activity calculated as [sample-inhibitor control].

### Mechanical testing of cartilage explants

The mechanical behaviour of individual cartilage explants was measured using an MTS, Bionix 100. A 2 mm diameter core was cut from the centre of each 5 mm diameter cartilage explant and a tare load of 0.01N applied to each explant which was then hydrated in culture media. The explants were subjected to a 20% uniaxial unconfined compressive strain (20%/min). This was followed by a stress relaxation period at constant 20% strain in which the load was recorded for a further 300 s. The load was recorded throughout the test at a sampling frequency of 60 Hz. Stress–strain and stress–time curves were generated for each specimen and the following mechanical properties of the cartilage determined:

$$Tangent\;Modulus\;(MPa)\;\;=\;\;\frac{\sigma_{\varepsilon \;=\;0.2}-\;\sigma_{\varepsilon \;=\;0.18}}{0.02}$$

$$Relaxation\;Modulus\;(MPa)\;\;=\;\;\frac{\sigma_{t\;=\;300s}}{0.2}$$

$$Percentage\;Relaxation\;(\%)\;\;=\;\;\frac{\sigma_{t\;=\;0s}-\;\sigma_{t\;=\;300s}}{\sigma_{t=0s}}$$

The relaxation half-life was calculated as the time from the start of the relaxation phase until stress reduced to half the peak value.

### Statistical analyses

The sample size for each experiment was chosen based on previous studies^5^^,^^32^ where analysis of cartilage degradation by biochemistry, immunohistochemistry and mechanical testing demonstrated that 6–8 samples/group is sufficient to detect a 25% difference in cartilage matrix catabolism at 80% power and a significance of *P* < 0.05. Data analysis was conducted using GraphPad Prism version 8 (GraphPad software, La Jolla California USA, www.graphpad.com). Normality testing (Kolmogorov Smirnov test) was performed to confirm that data exhibited Gaussian distribution. For data sets that were not normally distributed, namely cilia length data, Box Cox transformation (*λ* = 0.5) was performed prior to statistical analyses. Statistical significance was determined by *T*-Test, One-way, Two-way or Three-way analysis of variance (ANOVA) as appropriate with post-hoc Tukey's multiple comparisons performed to identify significant differences between groups. Statistically significant differences were determined based on a threshold of ∗ = *P* < 0.05, ∗∗ = *P* < 0.01 and ∗∗∗ = *P* = 0.001. Data is presented as mean ± standard deviation (SD) unless otherwise stated.

## Results

### TRPV4 activation is required for the anti-inflammatory effects of mechanical loading in isolated chondrocytes

**Fig. 1:**
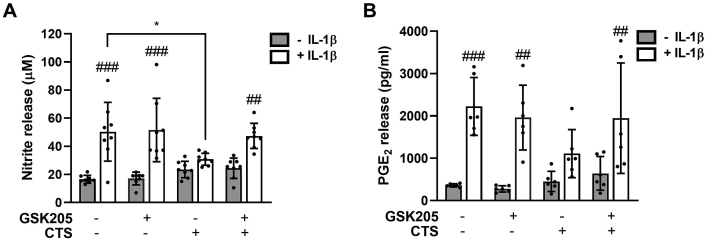
**Cyclic tensile strain inhibits IL-1β mediated NO and PGE2 release via a TRPV4 dependent pathway in isolated chondrocytes**. The TRPV4 antagonist, GSK205 (10 μM), abolishes the anti-inflammatory effects of mechanical loading (CTS, 10%, 0.33 Hz) at 1 ng/ml IL-1β on (A) nitrite and (B) PGE_2_ release at 24 h. Data represents mean ± SD for *n* = 6 wells per group using cells isolated from 2 different donors. Statistics: Three-way ANOVA and *post hoc* Tukey's test. # represents statistically significant difference between IL-1β treated and corresponding untreated cells.

IL-1β treatment (24 h) resulted in significant, dose-dependent release of NO and PGE_2_ (Fig. 1AB, (S)1AB). In response to 1 ng/ml IL-1β isolated chondrocytes exhibited a 3.04-fold increase in nitrite levels indicative of NO release [Fig. 1(A)], and a 4.84-fold increase in PGE_2_ release [Fig. 1(B)] which increased to 11.48- and 7.37-fold respectively in response to 10 ng/ml IL-1β (Fig. S1A-B). Consistent with previous studies^5^ this response was significantly reduced by mechanical loading in the form of CTS. IL-1β induced NO release was abolished by CTS such that there was no statistically significant effect at either 1 or 10 ng/ml (Fig. 1(A), (S)1A). PGE_2_ release was completely inhibited by CTS at 1 ng/ml IL-1β [Fig. 1(B)] but only partially suppressed at 10 ng/ml (Fig. S1B).

Simultaneous treatment with the TRPV4 antagonist GSK205 (10 μM) abolished the anti-inflammatory effects of mechanical loading (Fig. 1, (S)1). While GSK205 had no effect on NO or PGE_2_ release in unloaded cells with or without IL-1β, in loaded cells the IL-1β response was restored such that NO (Fig. 1(A) and S1A) and PGE_2_ release (Fig. 1(B), (S)1B) were significantly increased by IL-1β. Neither IL-1β nor GSK205 treatment in the presence or absence of CTS influenced TRPV4 protein levels (Fig. S10). These data indicate the anti-inflammatory effects of mechanical loading are mediated by TRPV4 activation.

### TRPV4 activation is required for the anti-inflammatory effects of hypo-osmotic loading in isolated chondrocytes and cartilage explants

**Fig. 2:**
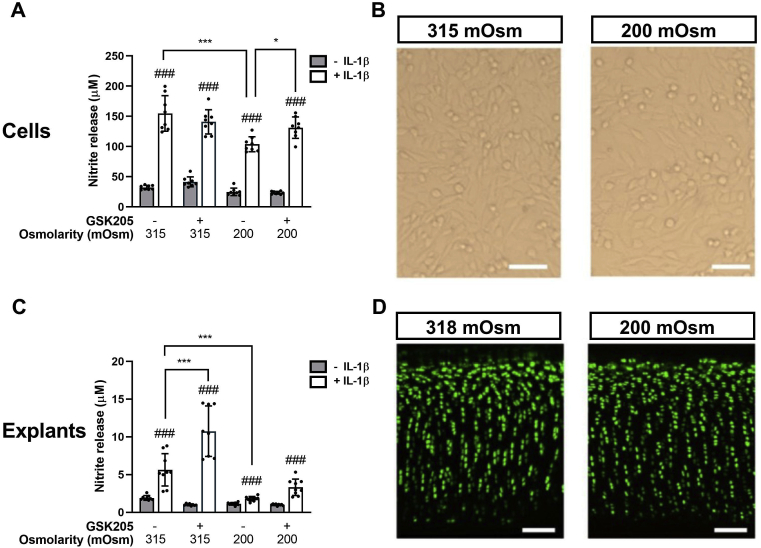
**Hypo-osmotic challenge inhibits IL-1β mediated NO release via a TRPV4 dependent pathway in isolated chondrocytes and cartilage explants**. The TRPV4 antagonist, GSK205, suppresses the anti-inflammatory effects of hypo-osmotic challenge. Nitrite levels measured in the media for (A) isolated cells and (C) cartilage explants in hypo- or iso-osmotic media. Chondrocytes and cartilage explants were treated with and without IL-1β (1 ng/ml) for 24 h and 12 d respectively with and without the TRPV4 inhibitor GSK205 (10 μM). Hypo-osmotic challenge had no effect on cell viability as determined by (B) bright filed images of isolated chondrocytes (D) confocal microscopy of explants stained with Calcein-AM (live cells, green) and ethidium homodimer (dead cells, red). Scale bar represents 100 μm. Data represents mean ± SD for *n* = 6 separate wells (A) or *n* = 8 separate explants (C) using cells/explants isolated from 2 different donors. Statistics: Three-way ANOVA and *post hoc* Tukey's test. # represents statistically significant difference between IL-1β treated and corresponding untreated cells.

Isolated chondrocytes were treated with hyper-osmotic media (400 mOsm), hypo-osmotic (200 mOsm) or iso-osmotic media (315 mOsm) for 24 h (Fig. 2AB, (S)2). Hyper-osmotic challenge had no significant effect on NO release, with or without IL-1β relative to the iso-osmotic control (Fig. S2, S3). By contrast, hypo-osmotic challenge significantly attenuated the pro-inflammatory response to IL-1β (1 ng/ml), such that the increase in NO release at 24 h was significantly reduced (*P* < 0.001, Fig. 2(A) S2A). Hypo-osmotic challenge had no apparent effect on cell viability compared to control conditions based on brightfield microscopy [Fig. 2(B)]. In the presence of GSK205, the anti-inflammatory effect of hypo-osmotic challenge on IL-1β induced NO release was completely inhibited by GSK205 such that the induction of NO release was not significantly different to control conditions [Fig. 2(A)]. In the absence of IL-1β, GSK205 also had no effect on baseline NO or PGE_2_ levels (Fig. S2).

In cartilage explants, hypo-osmotic challenge significantly reduced IL-1β induced NO release (Fig. 2(C), *P* < 0.001) such that there was no significant difference between IL-1β treated and untreated explants (Fig. 2(C) and S4A). In line with these findings hypo-osmotic challenge blocked the IL-1β mediated release of sGAG into the media, indicative of a reduction in extracellular matrix degradation (Fig. S4B). Chondrocyte viability was maintained throughout the experiment as determined by live/dead staining [Fig. 2(D)]. Consistent with isolated cells, hyper-osmotic challenge (400 mOsm, 12 d) had no effect on NO or sGAG release in the presence or absence of 1 ng/ml IL-1β (Fig. S4). GSK205 treatment restored IL-1β-induced NO release in hypo-osmotic media [Fig. 2(C)] thus blocking the anti-inflammatory effect of osmotic challenge. Interestingly, GSK205 further increased IL-1β-induced NO release in iso-osmotic, control media a response not seen in isolated cells [Fig. 2(C)]. Together these data indicate the anti-inflammatory effects of hypo-osmotic loading are also mediated by TRPV4 activation.

### TRPV4 activation is associated with altered primary cilia localisation and regulates cilia length

**Fig. 3:**
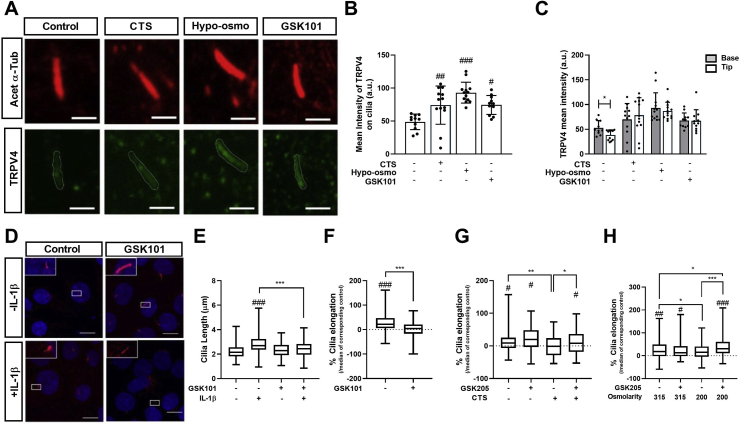
**TRPV4 activation increases cilia expression of TRPV4 and inhibits cilia elongation in response to IL-1β**. Primary articular chondrocytes were subjected to mechanical loading (CTS, 10%, 0.33 Hz), hypo-osmotic challenge (200 mOsm) or the TRPV4 agonist 1 nM GSK101 for 24 h (A) Representative maximum intensity projection of confocal images showing co-localisation of Acet-α-tubulin (Red) and TRPV4 (Green). Scale bar represents 1 μm. Pharmaceutical activation of TRPV4 increased the (B) mean intensity of TRPV4 labelling on primary cilia (*n* = 20–30 cilia) and (C) altered the distribution of TRPV4 on the cilium (*n* = 20–30 cilia) (D) Representative maximum intensity projection confocal microscopy images of isolated chondrocytes treated with ±1 ng/ml IL-1β ±1 nM GSK101 then labelled for acetylated α-tubulin (red) and counter stained with DAPI (blue). Scale bar represents 10 μm (E) Primary cilia length and (F) associated % elongation were measured at 24 h % cilia elongation results showing IL-1β induced change in cilia length for cells cultured (G) with and without mechanical loading and in (H) iso- and hypo-osmotic media. For % cilia elongation, data represents cilia length change in the presence of IL-1β (1 ng/ml, 24 h) normalised to median values in corresponding condition without IL-1β. Box plots are displayed as median, with error bars depicting min/max values (for E-H, *n* = 70–130 cilia). Statistics: One-way ANOVA (B) and Two-way ANOVA (D, E, G and H) with *post hoc* Tukey's test and *T*-test (C, F).

IL-1β induces primary cilia elongation in articular chondrocytes and mediates downstream catabolic NF-κB signalling through regulation of IFT^5^^,^^23^^,^^24^. We therefore examined the involvement of primary cilia in the anti-inflammatory mechanism of TRPV4 activation. TRPV4 cilia localisation was observed in isolated chondrocytes [Fig. 3(A)]. TRPV4 activation by mechanical loading, hypo-osmotic challenge or the TRPV4 agonist GSK101 (1 nM) increased TRPV4 cilia localisation while not significantly affecting protein expression, as shown by the increased mean intensity of ciliary TRPV4 (Fig. 3(B) and S10) and altered distribution profile of TRPV4 in proximal and distal regions of the axoneme [Fig. 3(C)] these data are suggestive of alterations in IFT.

In isolated chondrocytes, IL-1β (1 ng/ml) treatment for 24 h induced a significant increase in primary cilia length (*P* < 0.001) from a median value of 2.21–2.84 μm. This elongation was abolished by TRPV4 activation with GSK101 [Fig. 3(D)–(E)]. IL-1β mediated cilia elongation was also blocked by mechanical loading (CTS, 0–10%, 0.33 Hz, Fig. 3(G)) and hypo-osmotic challenge [Fig. 3(H)]. Inhibition of TRPV4 with GSK205 restored IL-1β mediated cilia elongation in the presence of both mechanical loading (*P* < 0.001, Fig. 3(G)), and hypo-osmotic challenge (*P* < 0.001, Fig. 3(H)). GSK101, had no effect on cilia length in iso-osmotic conditions with or without IL-1β [Fig. 3(E)]. GSK101 also had no effect on cilia prevalence for any of the treatment groups (Fig. S5A and D).

### TRPV4 activation inhibits inflammatory signalling in response to IL-1β through the regulation of HDAC6 and ciliary tubulin

**Fig. 4:**
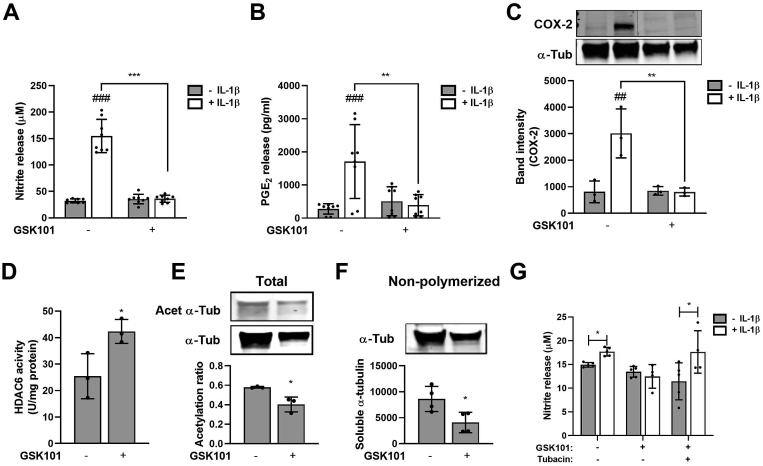
**TRPV4 activation abolishes IL-1β inflammatory signalling via HDAC6 activation**. Levels of (A) nitrite (B) PGE_2_ and (C) COX-2 expression associated with isolated chondrocytes ± IL-1β (1 ng/ml) in the presence or absence of GSK101 for 24 h. GSK101 promotes (D) HDAC6 activity, induces the (E) de-acetylation and (E) de-polymerization of α-tubulin, as measured by Western blot of acetylated α-tubulin (Acet α-Tub), α-tubulin (α-Tub) and non-polymerized α-tubulin. Full western blots can be found in supplementary figure S9 (G) HDAC6 inhibition with tubacin (500 nM) abolished the anti-inflammatory effect of GSK101 on NO release. Data represents mean ± SD for *n* = 6 (A, B, D and G) and *n* = 4 (C, E and F) Statistics: Two-way ANOVA and *post hoc* Tukey's test (A–C), *T*-test (D-G. # represents statistically significant difference between IL-1β treated and corresponding untreated cells.

We next examined whether direct pharmaceutical activation of TRPV4 would replicate the anti-inflammatory effect of mechanical and osmotic loading. IL-1β (1 ng/ml) induced the characteristic upregulation of NO and PGE_2_ release in isolated chondrocytes which was abolished by GSK101 [Fig. 4(A) and (B)]. Similarly IL-1β induced COX2 expression was abolished by GSK101 [Fig. 4(C)]. No effects on cell viability based on bright field microscopy (Fig. S6A) and deoxyribonucleic acid (DNA) content were observed although cells appeared to have a more rounded morphology particularly at high concentrations (Fig. S6B).

Previously, we identified a mechanistic role for HDAC6 activation and post-transcriptional tubulin modifications in the anti-inflammatory effect of mechanical loading^5^. Similarly, GSK101 resulted in significant upregulation of HDAC6 activity [Fig. 4(D)] suggesting TRPV4-mediated calcium signalling activates HDAC6. Consistent with this finding we observed significant tubulin deacetylation accompanied by a reduction in the pool of non-polymerized, soluble tubulin [Fig. 4(E)–(F)]. Furthermore, the HDAC6 specific inhibitor, tubacin (500 nM), restored IL-1β mediated stimulation of NO release in GSK101-treated cells [Fig. 4(G)]. These data suggest that GSK101 mimics the effects of mechanical loading on IL-1β inflammatory signalling, HDAC6 activation and tubulin modification.

### TRPV4 activation abolishes IL-1β mediated cartilage degradation and loss of mechanical properties

**Fig. 5:**
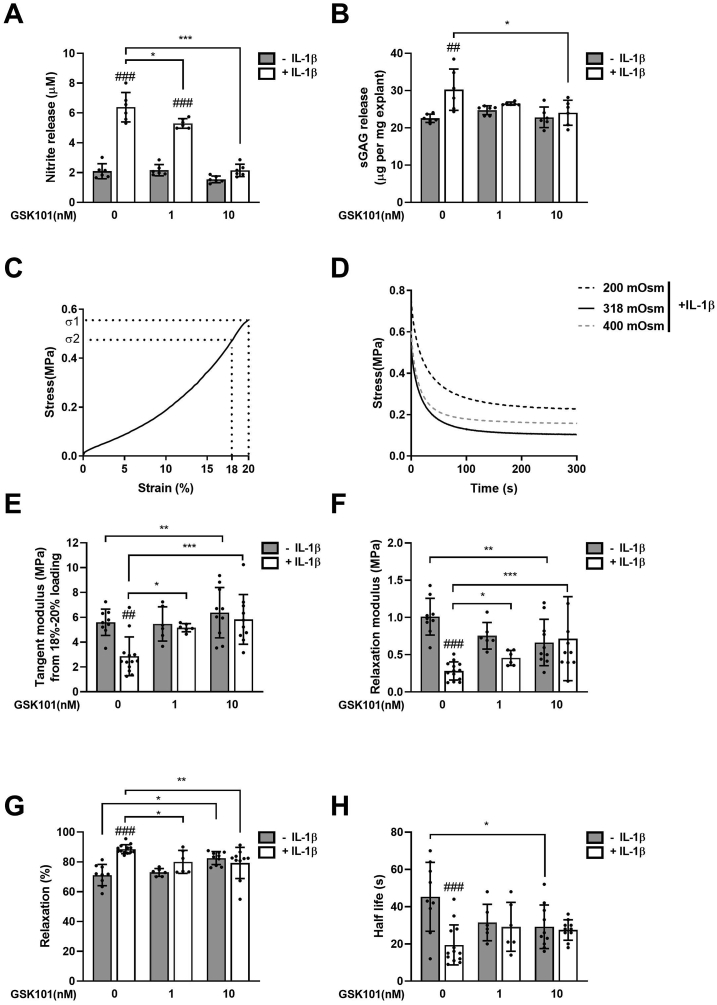
**TRPV4 activation suppresses IL-1β induced NO release, matrix degradation and loss of mechanical properties in cartilage explants**. Full-depth cartilage explants were treated with the TRPV4 agonist GSK101 (0, 1, 10 nM) in the presence or absence of 1 ng/ml IL-1β for 12 d. The nitrite (A) and sGAG (B) content of the culture media was measured and normalised to wet weight. Cartilage explants were compressed to obtain the stress–strain (C) and stress-relaxation (D) plots, for the calculation of cartilage mechanical properties. The responding mechanical properties of tangent modulus from 18 to 20% compression (E), relaxation modulus (F), percentage relaxation (G) and half-life (H). Data represents mean ± SD, *n* = 6–12 explants from 4 different donors. Statistics: Two-way ANOVA with *post hoc* Tukey's test. # represents statistically significant difference between IL-1β treated and corresponding untreated cells.

We next determined whether pharmaceutical activation of TRPV4 could prevent cartilage degradation and loss of mechanical properties. Cartilage explants were treated with IL-1β for up to 12 d in the presence of 1 nM or 10 nM GSK101. Cartilage explant viability was maintained at these experimental doses (Fig. S7). In response to IL-1β treatment, significant NO release was observed (Fig. 5(A), *P* < 0.001) indicative of activation of inflammatory signalling. This response was accompanied by significant sGAG release indicative of cartilage degradation (Fig. 5(B), *P* < 0.001).

We measured the viscoelastic properties of cartilage tissue using uniaxial unconfined compression to determine whether GSK101 could prevent the loss of mechanical properties induced by IL-1β. Cartilage explants exhibited a non-linear stress–strain graph represented by a tangent modulus of 15–20 MPa [Fig. 5(C)]. This was followed by characteristic viscoelastic stress relaxation at 20% strain [Fig. 5(D)] to a relaxation modulus of 2–3 MPa at 300 s representing 80% relaxation and a relaxation half-life of approximately 50 s [Fig. 5(E)–(H)]. IL-1β treatment resulted in dramatic loss of mechanical stiffness as shown by significant reductions in tangent modulus (*P* < 0.001, Fig. 5(E)) and relaxation modulus (*P* < 0.001, Fig. 5(F)), increased percentage relaxation (*P* < 0.001, Fig. 5(G)) and a reduction in half-life (*P* < 0.001, Fig. 5(H)).

GSK10 significantly inhibited the cumulative release of NO from cartilage explants in response to IL-1β treatment (*P* < 0.001, Fig. 5(A)). Similarly the cumulative release of sGAG was significantly reduced (*P* < 0.001) and loss of mechanical properties in response to IL-1β abolished, such that there was no significant difference in any of the biomechanical parameters with and without IL-1β.

## Discussion

This study demonstrates that TRPV4 plays an important mechanistic role in the anti-inflammatory effect of mechanical stimulation. TRPV4 inhibition restores IL-1β mediated pro-inflammatory signalling in the presence of both mechanical and osmotic loading. Conversely, TRPV4 activation by GSK101 blocked the release of pro-inflammatory mediators in the absence of load in isolated cells and prevented cartilage degradation and loss of mechanical properties in an explant model. TRPV4 is activated by mechanical stimulation in the form of CTS or osmotic challenge and functions upstream of HDAC6 to modulate tubulin acetylation and polymerization which regulates IFT thereby suppressing IFT-dependent IL-1β signalling.

TRPV4 is expressed in bone marrow stem cells, osteoblasts, osteoclasts and chondrocytes, and is required for skeletal development^10^^,^^33^. TRPV4 belongs to the TRP superfamily which mediate cellular responses to a variety of environmental stimuli including heat^34^, cell swelling^35^, hypo-osmolality^18^^,^^36^ and mechanical loading^7^^,^^9^ and results in elevated levels of intracellular Ca^2+^. Thus, TRPV4 is required for mechanotransduction. It promotes chondrocyte matrix production in response to dynamic compression^7^, mediates the fluid shear induced osteogenic response in stem cells^9^ and shear stress induced vasodilatation in endothelial cells^8^.

In other tissues, TRPV4 activation is mostly reported to be pro-inflammatory. In airway epithelial cells, TRPV4 activates NF-κB signalling promoting progression of lung fibrosis^37^. Endogenous TRP channel agonists are detected in a lung injury model while TRPV4 inhibition suppresses acid-induced pulmonary inflammation^38^. TRPV4 antagonists have been used to treat sepsis in mice by reducing production of TNF-α, IL-1 and IL-6^16^. Moreover loss of TRPV4 function suppresses inflammatory fibrosis in mouse corneas^39^. However, Xu *et al.* report that GSK101 prevents vascular inflammation and atherosclerosis, associated with inhibition of NO synthase and mitogen-activated protein (MAPK) signalling^14^. TRPV4 is also well-established to mediate inflammatory hyperalgesia (see review^40^) and is regarded as a promising target for novel analgesics.

Consistent with our findings, pharmaceutical activation of TRPV4 has been shown to suppress NO release induced by lipopolysaccharide (LPS) in rat temporomandibular chondrocytes, whereas TRPV4 inhibition aggravates the inflammatory response to LPS^13^. Clark *et al.* report that TRPV4 deficiency induces inflammation and disrupts cartilage matrix homeostasis. As such, TRPV4^−/−^ mice exhibit a severe sex-dependent osteoarthritis (male mice are more susceptible) while the isolated chondrocytes fail to increase Ca^2+^ influx in response to hypo-osmotic challenge^11^. These mice exhibit a more severe obesity-induced osteoarthritis, compared to wild-type mice^12^. However other studies report osmotic challenge to be a pro-inflammatory signal. Hubert *et al*. observed induction of IL-8 in response to both hyper and hypo-osmotic stress^41^ while hypo-osmotic stimulation of TRPV4 promoted PGE_2_ release in porcine chondrocytes^18^ and the expression of IL-1β and IL-6 in bovine intervertebral disc cells^36^, suggesting a pro-inflammatory role of TRPV4. We did observe a mild, transient increase in NO release in this study at 3 h hypo-osmotic challenge however this had resolved and was not significantly different to the control at 24 h (Fig. S8). Interestingly we observed dose-dependent cytotoxicity of GSK101 with extended explant culture at concentrations above 10 nM (Fig. S7). Low concentrations of GSK101 elicit multiple short peaks of Ca^2+^ signalling, which is more physiological compared with the large, sustained peaks observed at higher concentrations which might explain this^42^. These observations suggest perhaps that only moderate, short-term modulation of TRPV4 will be chondroprotective.

Servin-Vences *et al*. suggest TRPV4 mechanosensitivity is dependent upon the type of stimulus applied^6^. Our data supports this hypothesis, complete abolition of NO release in response to IL-1β was observed following application of cyclic tensile strain (Fig. 1), while hypo-osmotic challenge merely attenuated the response (Fig. 2) suggesting TRPV4 activation may be regulated distinct mechanisms and to different extents. Vriens *et al*. report that TRPV4 activation in response to cell swelling is dependent upon arachidonic acid release^43^ whereas Servin-Vences *et al*. suggest direct channel gating occurs in response to membrane deflection^6^.

The mechanosensitive ion channel PIEZO1 reportedly induces TRPV4 channel opening^44^. PIEZO1 is activated chondrocytes following injurious loading and is suggested to play a greater role in chondroprotection than TRPV4^6^^,^^45^. It is possible the more pronounced anti-inflammatory effects of CTS observed in this study are the result of further TRPV4 activation downstream of this channel, which could be explored in future studies. However, while activation of PIEZO1 reportedly influences ciliogenesis^46^ studies in osteocytes indicate that it does not interact with TRPV4 in the cilium^47^.

TRPV4 cilia localisation was observed with greater localisation evident at to the ciliary base [Fig. 3(C)]. TRPV4 activation altered this distribution such that localisation to the base or tip of the axoneme was not significantly different indicative of altered protein trafficking/IFT [Fig. 3(C)]. TRPV4 activation is coupled with translocation of TRPV4 to plasma membrane^48^, in this study we observed increased TRPV4 labelling in the ciliary membrane [Fig. 3AB]. Chemical deletion of primary cilia with chloral hydrate fully abolishes Ca^2+^ signalling in response to TRPV4 activation^18^ thus increased ciliary TRPV4 may be important for signalling.

HDAC6 is enriched within primary cilia catalysing tubulin de-acetylation and polymerization to regulate cilia resorption^27^, ^28^, ^29^. In this study, mechanical, hypo-osmotic and pharmaceutical activation of TRPV4 blocked cilia elongation in response to IL-1β. IFT and cilia elongation is required for IL-1β mediated inflammatory signalling and downstream NF-κB signalling^5^^,^^23^^,^^24^, therefore we suggest the anti-inflammatory effects of TRPV4 activation regulate IFT and associated signalling via HDAC6 dependent modulation of ciliary tubulin. Previous studies demonstrate that GSK101 activates Ca^2+^ signalling in isolated chondrocytes^6^^,^^17^^,^^49^, while GSK205 inhibits this response and blocks Ca^2+^ signalling in response to mechanical or osmotic loading^7^^,^^18^^,^^50^. While Ca^2+^ signalling was not assessed in the current study, we hypothesise that Ca^2+^ levels may regulate HDAC activity through activation of upstream kinases such as Ca^2+^/Calmodulin dependent kinase (CaMK), protein kinase D (PKD) and Aurora A kinase-dependent (AURKA)^27^, ^28^, ^29^^,^^51^, ^52^, ^53^, ^54^. Studies suggest TRPV4 stimulation with GSK101 promotes extracellular-signal-related kinase (ERK)/MAPK signalling in lung epithelial cells and cancer cells^55^ and PKC activity in endothelial cells^56^ which phosphorylate HDAC6 resulting in increased deacetylation activity^57^^,^^58^. Indeed, increased HDAC6 activity was observed in response to GSK101 [Fig. 4(D)].

In conclusion, this study demonstrates a role for TRPV4 activation in the anti-inflammatory mechanism of loading. In addition to providing new mechanistic understanding of this pathway, this study identifies TRPV4 as a potential therapeutic target and demonstrates that pharmaceutical activation of this protein could regulate inflammation and other IFT-dependent pathways involved in cartilage disease.

## Contributions

All authors aided in revising this manuscript for intellectual content and approved the final version to be published.

Study design: Su Fu, Clare L Thompson, Martin M Knight.

Data acquisition: Su Fu, Clare L Thompson, Sheetal Inamdar, Huan Meng.

Data analysis and interpretation: Su Fu, Clare L Thompson, Sheetal Inamdar, Wen Wang, Himadri Gupta, Martin M Knight.

## Conflict of interest

The authors have no competing interests.

## Funding sources

Su Fu and Huan Meng are funded by the 10.13039/501100004543China Scholarship Council for his PhD studies at 10.13039/100009148Queen Mary University of London. Dr Clare 10.13039/100004686Thompson is supported by a project grant from the UK 10.13039/501100000265Medical Research Council (No: MR/L002876/1, PI: Knight). Sheetal Inamdar is supported by a project grant from the Biotechnology and Biomedical Sciences Research Council (No: BB/R003610/1, PI: Gupta).
