# Supplementary material for: Activation of TRPV4 by mechanical, osmotic or pharmaceutical stimulation is anti-inflammatory blocking IL-1β mediated articular cartilage matrix destruction
Source: Osteoarthritis Cartilage. 2021 Jan;29(1):89–99. doi: 10.1016/j.joca.2020.08.002 (PMC7799379; doi:10.1016/j.joca.2020.08.002)
Supplement: Supplementary file 1 — Multimedia component 1 [file mmc1.docx]

Activation of TRPV4 by mechanical, osmotic or pharmaceutical stimulation is anti-inflammatory blocking IL-1β mediated articular cartilage matrix destruction.

Su Fu, Sheetal Inamdar, Himadri Gupta, Wen Wang, Clare L Thompson*, Martin M Knight

Institute of Bioengineering, School of Engineering and Materials Science, Queen Mary University of London

Supplementary figures.

**Figure S1. Cyclic tensile strain inhibits NO and PGE_2_ release in response to 10ng/ml IL-1β via a TRPV4 dependent pathway in isolated chondrocytes.** The TRPV4 antagonist of GSK205 (10μM) also abolished the anti-inflammatory effect of CTS at 10ng/ml IL-1β attenuating nitrite (A) and PGE2 (B) release following treatment for 24hrs. Data represents mean ±SD, n=6 replicates from 2 different donors. Statistics: Three-way ANOVA and post Tukey’s test.

**Fig S2. Hyper-osmotic challenge (400 mOsm, 24hrs) has no effect on either NO or PGE_2_ release from isolated bovine chondrocytes in the presence or absence of IL-1β (1ng/ml) in contrast to the anti-inflammatory effects of hypo-osmotic challenge (200 mOsm).** The levels of nitrite (A) and PGE_2_ (B) release for chondrocytes cultured in 200, 315 or 400mOsm with and without 1ng/ml IL-1β for 24 hrs. Data represents mean ± SD where n=14. Statistics: Two-way ANOVA and Tukey’s test, ‘#’ refers to the difference between -IL-1β and + IL-1β.

**Fig S3. Hypo-osmotic challenge (24hrs) reduces pro-inflammatory signalling in a murine chondrocyte cell line.** Hyper-osmotic challenge has no effect compared to iso-osmotic conditions. The levels of nitrite (A) for murine chondrocytes cultured in 200, 315 or 400mOsm with and without 1ng/ml IL-1β for 24 hrs. Data represents mean ± SD, n=6. Statistics: Two-way ANOVA and Tukey’s test, ‘#’ refers to the difference between -IL-1β and + IL-1β.

**Fig S4. Hyper-osmotic challenge (400 mOsm, 12 days) has no effect on NO or sGAG release from cartilage explants in the presence or absence of IL-1β (1ng/ml) in contrast to the anti-inflammatory effects of hypo-osmotic challenge (200 mOsm).** Full depth cartilage explants were cultured in osmotically active media (200mOsm, 318mOsm or 400mOsm), in the presence or absence of 1ng/ml IL-1β for 12 days. The cumulative release levels of nitrite (A) and sGAG (B) were measured. # means difference caused by IL-1β. Data represents mean ± SD, n=18 from two separate experiments (n=8 from 4 donors and n=10 from 6 donors, respectively). Statistics: Two-way ANOVA and Tukey’s test, ‘#’ refers to the difference between -IL-1β and + IL-1β, ‘+’ difference relative to–IL-1β control at 200mOsm and ‘*’ as shown.

##

**Fig S5. Corresponding cilia prevalence in Fig 3.** The corresponding cili**a prevalence results in Figure 3** C-E showing that either GSK101, GSK205, mechanical loading or hypo-osmotic challenge did not influence the cilia prevalence. Chi-square test and Fisher’s exact p value test. n= approximately 150 cells.


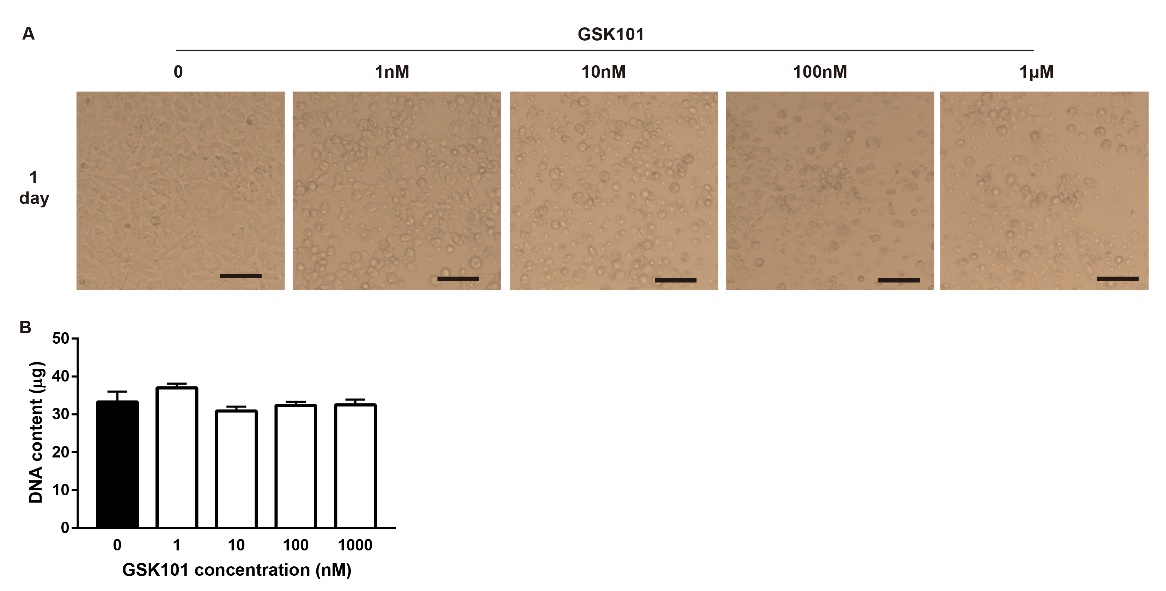


**Fig S6. GSK101 treatment in isolated chondrocytes for 24h does not influence cell viability.** The influence of GSK101 on cell viability in cultured chondrocytes for 24 hours, showing by (A) bright field microscopy and (B) DNA content measurement after PBS washing. Black bar represents 100 µm. n= 6 separate wells (B). Statistics: One-way ANOVA and Tukey’s post hoc test.


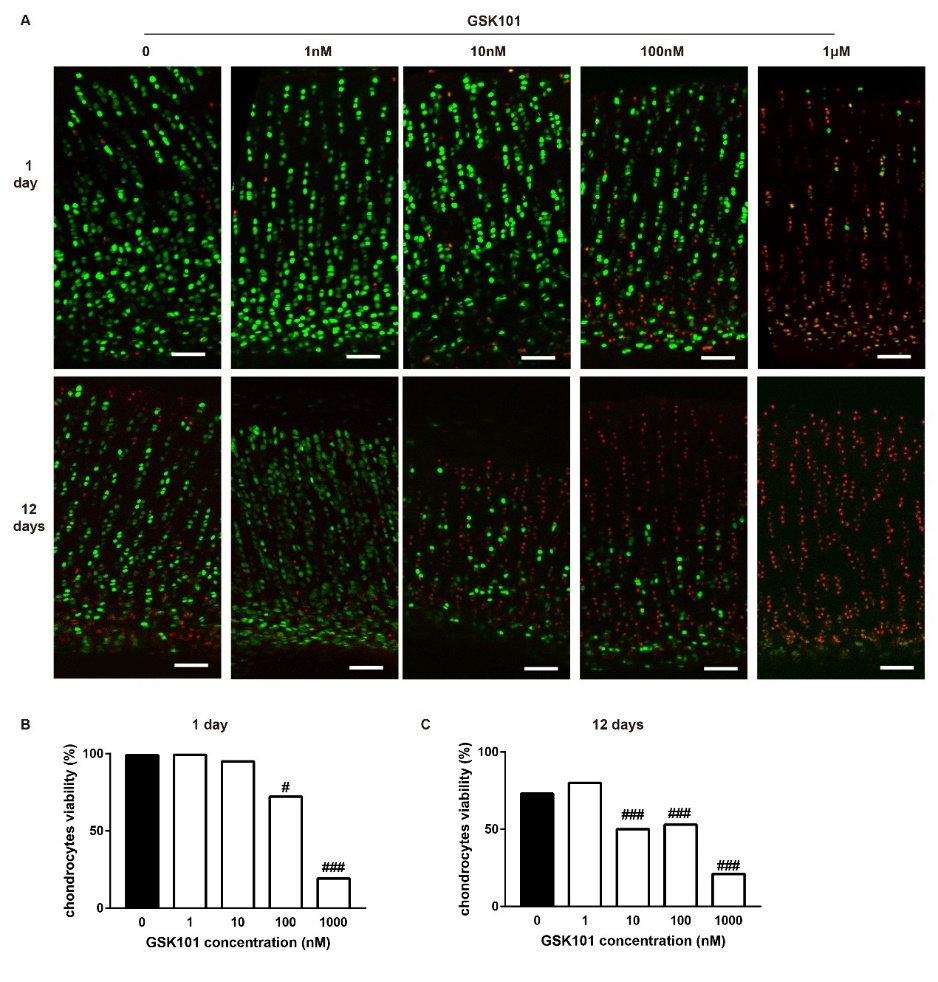

**Fig S7. GSK101 treatment reduces cell viability in full-depth cartilage explants in a dose dependent manner.** Articular cartilage explants were cultured in the absence or presence of TRPV4 agonist, GSK101 (1, 10, 100 or 1000nM) for 24 hrs and 12 days. (A) Confocal microscopy of explants stained with Calcein-AM (live cells, green) and ethidium homodimer (dead cells, red). Scale bar represents 100 µm. Corresponding percentage numbers of live cells within cartilage for 24 hrs (B) and 12 days (C). # means difference in cell viability caused by GSK101. Data represent mean ±SD, n>200 cells. Statistics: One-way ANOVA and Tukey’s test.

**Fig S8. In the absence of IL-1β, hypo-osmotic challenge (200 mOsm) induces a mild transient pro-inflammatory response**. (A) NO and (B) PGE_2_ release in isolated chondrocytes compared to iso-osmotic controls (315 mOsm). Data points represent mean ±SD for n=6 replicate. Statistically significant differences were indicated relative to iso-osmotic conditions at the same time point (* p<0.05, *** p<0.001).


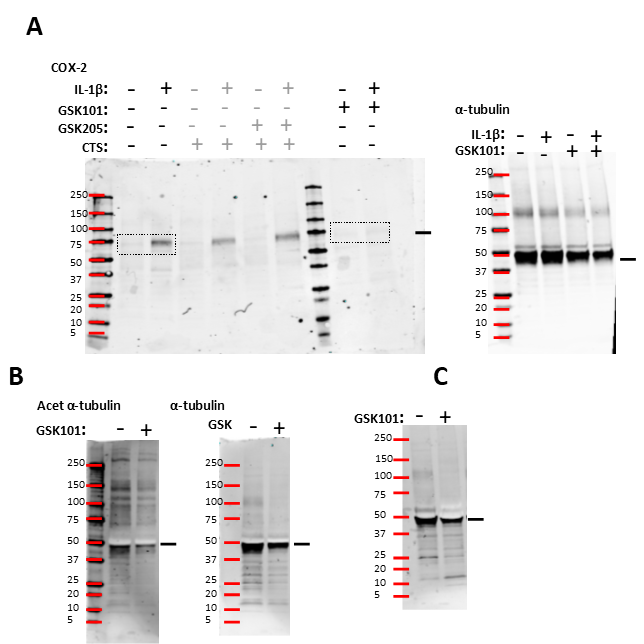


**Fig S9. Uncropped western blots from figure 4.** (A) COX-2 and α-tubulin western blots from figure 4A. (B) Total acetylated α-tubulin and α-tubulin western blot from figure 4E. (C) Non-polymerized α-tubulin western blot from figure 4F.


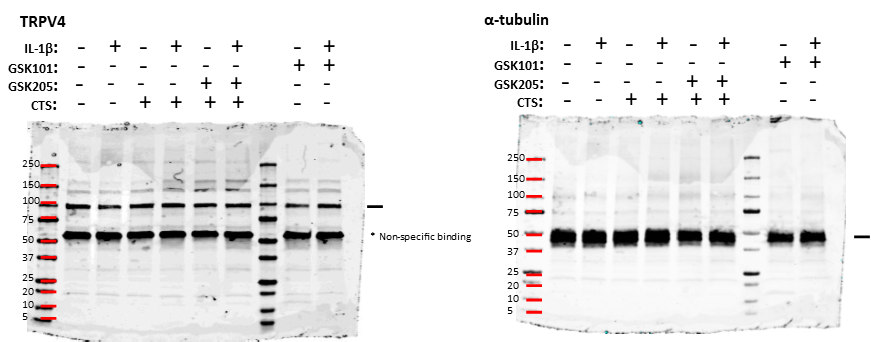


**FigS10. TRPV4 protein expression was not altered in response to IL-1, GSK205 or GSK101 stimulation.**

TRPV4 (100kDa) and α-tubulin (45kDa) western blot, appropriately sized bands are marked. Samples were treated and loaded for 24hrs, 10% cyclic tensile strain at 0.33Hz.
